# Comparative Evaluation of the Sensory Qualities of Refined and Wholegrain Rice as Ingredients within Mixed Dishes

**DOI:** 10.3390/nu16131984

**Published:** 2024-06-21

**Authors:** Andreia Da Graça, Foyeke Teinye-Boyle, Iain A. Brownlee

**Affiliations:** Faculty of Health and Life Sciences, Northumbria University, Newcastle-upon-Tyne NE1 8ST, UK; dagracaandreia.12@gmail.com (A.D.G.); foyikstoba@gmail.com (F.T.-B.)

**Keywords:** wholegrain food, whole grains, taste tests, sensory evaluation

## Abstract

Low wholegrain food consumption is a leading dietary risk for avoidable morbidity and mortality globally, with limited sensory acceptability suggested to be a challenge for changing behaviour. This study aimed to evaluate the sensory acceptability of both wholegrain (brown) and refined (white) rice in common preparations. Four brown- and white-rice-containing dishes (Garlic Rice, Rice and Beans, Jollof Rice, and Rice Pudding) were tested. Quantitative (five-point scales) and qualitative (open question responses) sensory information were collected for dish appearance, aroma, taste, and texture. All four characteristics were scored equally acceptable in Rice and Beans and Rice Pudding (*p* > 0.05) between paired comparisons for brown and white rice. Scores were significantly lower for all characteristics for Jollof Rice (*p* ≤ 0.002), and lower for Aroma (median (lower quartile–upper quartile)) for brown (3.5 (3–4)) vs. white rice (4 (4–5)), *p* = 0.006). Appearance (brown (3 (3–4)) vs. white rice (4 (3.25–5)), *p* = 0.012), and Texture (brown 3 (2.25–4) vs. white rice (4 (4–5)), *p* < 0.001) for Garlic Rice. Familiarity and appealingness were qualitative themes aligned with the higher acceptability of white-rice-containing dishes. Certain dishes appear to mask key negative sensory attributes of wholegrain foods, possibly representing a means to increase wholegrain ingredient acceptability, thereby potentially improving individual/population-level intake.

## 1. Introduction

The term “wholegrain foods” has multiple definitions [1,2,3], but broadly refers to foods containing appreciable levels of intact or physically processed (including flakes and flour), edible components of the caryopsis (endosperm, bran, and germ) from the *Poaceae* family (cereal grains), alongside some pseudocereals. Wholegrain foods are higher in fibre than their refined grain equivalents and usually have a more positive overall compositional profile [4].

Wholegrain foods are referred to in many food-based dietary guidelines around the world, sometimes being defined as a “core” food group [5,6]. Global intake of whole grains is well below levels that have previously been associated with reduced non-communicable disease risk. Low wholegrain food intake has been suggested to be the major dietary risk factor for all-cause morbidity and, behind excess sodium intake, the second biggest risk for all-cause mortality [7]. As such, increasing wholegrain food intake at a population or global community level is a key challenge in public health nutrition.

Despite being a rational target to improve public health through modifying dietary behaviour, there appear to be multiple, key barriers to wholegrain food consumption. Previous studies on consumer perceptions of wholegrain foods have highlighted issues like availability, price, cooking time, and family unit preferences that may all affect whether wholegrain or refined grain options are chosen, with perhaps the most consistent finding being the expectation that wholegrain foods will have inferior taste and texture in comparison to refined grain analogues [8,9,10,11,12,13,14]. The sensory appeal of all foods plays a major role in consumer choice and decision-making [15,16]. However, very few studies to date have actually measured the sensory attributes of wholegrain versus refined grain products [17]. There has also been limited consideration as to how acceptable wholegrain ingredients in complex dishes are.

Rice is not only the most commonly consumed grain in the world [18], but also the food commodity that provides the greatest amount of dietary energy to the global population [19]. Rice is a versatile ingredient that can be used in multiple dishes [20], acting as a staple for most regions of the world [18]. As such, it represents a rational ingredient to compare acceptability of refined and wholegrain ingredient alternatives.

The aim of the current study was to assess the sensory appeal of dishes containing brown (whole grain) and white (refined) rice. Multiple rice preparations were chosen to partially or fully mask the visual attributes of the rice ingredient.

## 2. Materials and Methods

### 2.1. Ethical Approval and Study Design

Ethical approval for this sensory analysis study was granted on 20 February 2023 (ID: 1609) by Northumbria University Faculty of Health and Life Sciences Research Ethics Committee, with additional updates (to expand dishes included and total participant numbers from 22 June 2023). Participants were recruited by word of mouth locally. Participants had to be adults, i.e., 18 years or above, and were requested to confirm that they were not allergic or intolerant to the ingredients used to prepare the study foods.

### 2.2. Sensory Analysis

Following informed consent, participants were recruited to attend one sensory analysis, with some participants attending tests for multiple different rice dishes (see below). Each sensory visit day included a comparison of a single dish with a brown and white rice version served in random order. To protect anonymity and minimize risk of bias during data handling, all participant questionnaire responses were randomly coded.

Participants were served study foods in sensory booths, as per standard sensory evaluation [21]. Participants were not informed whether they were receiving the brown rice or white rice version of a dish in all instances, although this will likely have been visually apparent (e.g., for garlic rice).

All ingredients were procured from a national supermarket chain (Sainsbury’s, London, UK). The white and brown rice variety used for all dishes was “easy cook”. All dishes were prepared in test kitchens following staff induction for good food hygiene and research practice. Four dishes containing rice were provided to participants at separate sensory visits. Further details on recipes and ingredients are provided in Appendix A.1. These dishes were chosen based on the differences in visual appearance and taste profiles that might differentially affect acceptability of the brown and white rice [22]. The rice dishes chosen also aimed to capture a variety of ways in which rice might be used as an ingredient across multiple cultures [18,19,20]. To ensure inclusivity, all dishes were prepared using vegan ingredients. The four dishes were:

1. Garlic Rice—contains plain, boiled rice and minimal additions for flavour only.
2. Rice and Beans—a fried, savoury dish also containing pulses. The colour of additions and from frying masked the rice appearance [23].
3. Jollof Rice—a stewed, savoury dish with a tomato base. Again, the ingredients (particularly tomato) masked the appearance of the rice ingredient [24].
4. Rice Pudding—a sweet dish prepared by cooking rice with fat and milk (or milk alternatives) [25]. Brown sugar was used to mask the appearance of the rice ingredient.

For each dish, a standardized questionnaire was used to collect qualitative and quantitative data (see Appendix A.2) for both the refined rice and brown rice equivalents. As the study design required comparison of paired samples, a total sample size of *n* = 30 was chosen for each dish, assuming the potential for respondents to not try both the brown and white rice alternatives for a given dish. Based on the potential to observe a medium effect size, *n* = 26 participants completing the study provided an 80% chance of seeing a difference in white and brown rice acceptability if one existed [26]. We recruited higher numbers during data collection (up to 32 participants per pairwise comparison) in case submitted data were subsequently found to be unusable. The questionnaire was designed based on evaluating the most common organoleptic characteristics noted to be potential barriers for wholegrain food acceptability [8,9,10,11,12,13,14]. We used a five-point scale linked to the acceptability of each characteristic (appearance, aroma, taste, and texture), ranging from “Like very much” to “Dislike very much”. A further option was provided if participants were unwilling to try the dish, although this was not used by any study participant. This choice was based on preliminary methods developed in related research.

Foods were prepared immediately prior to each taste test. Food samples were presented to participants in a standard serving size using a 60 mL cup, served on standard white paper plates with the same utensils for consistency throughout tests for whole grain and refined grain equivalents of each of the four dishes.

Quantitative data were analysed using SPSS (version 26.0, IBM, Armonk, NY, USA). Due to the nature of 5-point scale data, non-parametric tests were used to compare paired data on acceptability of white rice dishes versus brown rice dishes for each of the four recipes across the four sensory parameters. A *p*-value of less than 0.05 was considered statistically significant. A ranking value of 5 was aligned with the choice “Like very much” and 1 for “Dislike very much”.

## 3. Results

A total of 93 participants (48 females, 43 males, 2 did not respond to question on sex) took part in 119 sensory visits across the four products. A summary of demographic and background data is presented in Table 1 below. Participants in all groups were similar in sex, age range, frequency of rice consumption, and the types of rice they habitually consumed.

Data from sensory panels are collated in Table 2. In general, white rice equivalents were rated more positively across most characteristics for most dishes. Brown rice alternatives of the Jollof Rice scored statistically lower across all four characteristics (appearance white rice median (IQR) vs. brown rice median (IQR) of 5 (4–5) vs. 4 (3–4.75), *p* = 0.002, aroma (4 (4–5) vs. 4 (3–4), *p* < 0.001), taste (4 (4–5) vs. 3 (3–4), *p* < 0.001), and texture (4 (4–5) vs. 3 (2–4), *p* = 0.001). All characteristics of the Rice and Beans dish were statistically comparable (*p* > 0.05) for white rice and brown rice alternatives.

Qualitative responses to open questions tended to be concise (a few words long from most participants). A series of common themes were linked to the acceptability of the alternatives for all rice-based dishes. From these, a series of common themes were noted in relation to acceptability of brown and white rice alternatives. These are summarized below in Table 3 in relation to appearance, aroma, taste, and texture. A consistent, positive theme noted for the white rice alternatives was the concept of familiarity, which was noted across all characteristics. Negative concepts on the colour of brown rice were shared, particularly in relation to the Garlic Rice dish, which somewhat aligns with scoring of appearance (see Table 2 above). Positive, negative, and neutral responses were received in relation to the comparable acceptability of brown rice versions of dishes (particularly Rice and Beans) in comparison to white rice alternatives. Themes shared relating to the appearance and texture of brown rice alternatives tended to include divergent views on preferability in relation to the white rice alternatives, although themes for these characteristics for white rice samples consistently suggested favourable attitudes.

## 4. Discussion

The overarching findings of these sensory analyses suggest that white rice is preferred to brown rice, although negative perceptions or experience of brown rice consumption could be masked completely within specific mixed dishes. Within those tested here, the Rice and Beans and Rice Pudding dishes were equally acceptable for brown rice and white rice alternatives. Many countries of the world currently have focused and concerted public health messages around increasing wholegrain food consumption [2,5,27,28]. These findings represent a simple, acceptable, and effective way in which such messages could be translated to support increased wholegrain food intake at an individual or population level. While the range of culinary uses of rice is not exhaustive within this study, the authors feel that the chosen dishes capture a range of preparations that have cultural significance across many parts of the world. It should be noted that specific dishes could therefore support more frequent intake of wholegrain foods. Incorporation of wholegrain ingredients into dishes could also either drive dietary habits towards intake guidelines (e.g., increased intake of pulses, vegetables, and total fibre in the case of Rice and Beans) or away from them (e.g., increased intake of free/added/total sugars in the case of Rice Pudding).

Three out of the four characteristics tested (appearance, aroma, and texture—see Table 2) scored statistically worse for the brown rice alternative than the white rice alternative for Garlic Rice. This dish was chosen as a flavoured item where the visual differences of the two types of rice would be apparent. A wide range of previous work has suggested that visual cues are a key driver for acceptance of grain foods [10,11,29,30], including rice [31,32].

Perhaps surprisingly, Jollof Rice made with brown rice scored statistically worse for all characteristics tested (see Table 3), including appearance. This dish was chosen for its initial testing due to the assumption that the colour of the rice used would be masked by tomatoes/tomato paste. It is possible that participants may have picked up on other visual cues, such as grain size or shape that influenced their perceptions of the final dish [33,34].

Within the current project, we only evaluated the wider construct of “appearance”. Further detail of the constructs that might relate to this characteristic were not asked within quantitative questions, but were partially informed by qualitative responses for preference (see Table 3 and discussion below). Interestingly, appearance also seemed to provide the greatest divergence in individual responses in terms of the two rice types, with some participants tending to favour the visual appeal of either brown or white rice, or suggesting that the two dishes were of equal appeal regardless of the rice type used.

We did not see clear divergence in themes raised for the different dishes chosen, with the more obvious separation noted in relation to the type of rice used (see Table 3). The overarching themes that were noted to align with sensory acceptability of white rice dishes were familiarity and perceived appealingness. In most parts of the world, wholegrain food options tend to be viewed as a secondary option rather than the preferable alternative [8,9]. The cultural norm of the consumption of refined grain options may be a key factor that influences conscious or unconscious decisions based on the appearance of wholegrain foods [35]. While public–private partnership approaches in Denmark successfully increased national wholegrain intake, it did so with average, pre-intervention intake in children and adults, which was much higher than most other parts of the world [27]. Altering food choice in populations where whole grains are not seen as a “normal” or standard choice of staple may have additional challenges, but should nonetheless be considered as a rational strategy of reducing risk of non-communicable disease. Terminology used to define white rice options as the most appealing and brown rice options as the least appealing somewhat aligns with that noted in previous works [11,22,31].

Rice was chosen as a logical ingredient for initial evaluation within this study due to how frequently it is consumed globally [18,19]. Brown rice is specifically mentioned in food-based dietary guidelines in many countries around the world; brown rice is a wholegrain food ingredient that is consumed in a “minimally-processed” state compared to other wholegrain food choices as eaten [36]. However, rice has a relatively low proportion of bran and germ as compared to other grain sources, so switching from white rice to brown rice may have less overall impact on immediate nutrient intake and possible lifelong health trajectory than other wholegrain foods [37]. Nonetheless, we believe that this paired, comparative approach could be tested across multiple different dishes, using different whole and refined grain sources, with different starting ingredients (e.g., flakes, flour, or more complex, processed staple items like pasta and noodles) or indeed differing amounts.

The approach here provides direct comparison of preferences to dishes containing wholegrain and refined ingredients. However, it must be noted that these findings relate solely to the acute, comparative acceptability. As such, they model the opinion of a single meal occasion and not the behaviour change over a more prolonged period of time. Preference at a single meal occasion has been shown to be modified by context and situation [11,29,32,38], and there is a potential that findings collected in a sensory suite are unlikely to effectively represent choice in a real-world setting [39,40,41]. However, previous work has also suggested that trial acceptance of wholegrain foods is a key barrier that must be overcome before longer-term acceptance is a possibility. To date, very few studies have looked at how long-term behaviour change might be affected by trial or exposure to whole grains, but these studies suggest a positive effect on wholegrain food acceptance [10,11,42,43].

Within the current approach, we defined the characteristics of interest based on those noted in previous literature and involved untrained participants to help model acceptability in the wider population. While this approach aligns well with the current research question, the authors acknowledge a number of ways that future work could consider improving on the current study design. Firstly, future studies can use a less hypothesis-driven approach to consider factors that might impact rice acceptability. For example, a Quantitative Descriptive Analysis approach would design a series of experiments for trained panellists to uncover specific factors that might drive product acceptability [44]. Such an approach could provide a more standardized and exploratory approach in a manner frequently used in product development. However, such a study design would appear less likely to model the general population. The authors believe that the inclusion of untrained panellists is most rational in this and other studies, where the outcomes relate to changing common dietary habits at a population level.

Unlike previous works [22,45], we have also not collected detail on overall rating/liking of the products involved. Collection of such information would have supported better understanding of which characteristics were most important in brown rice acceptability. Based on the statistical differences noted in the current study, it seems apparent that texture (which was statistically lower for three out of four wholegrain dishes versus their refined equivalents) may be the most relevant characteristic, and one that should be considered in the formulation of any future, novel wholegrain food products in greater detail.

The 5-point scale we used appears to have been sensitive enough to detect large differences in acceptability between the alternative dishes. However, previous studies had different approaches to collect acceptability data (e.g., 9-point Likert scales or visual analogue scales) that had a greater chance to detect minimal differences between two different dishes [22,45]. Such approaches would appear to be more rational for use in future studies looking at smaller changes in recipes, such as modifying the proportion of wholegrain and refined grain ingredients included.

With the current study, familiarity was noted as a key attribute for rice acceptance. The approach undertaken here lacked objective assessment of rice consumption habits beyond rice types consumed and estimated frequency of rice consumption (see Table 1). We also did not evaluate previous experience of the dishes used in the current study. There is potential that the most familiar products to individuals were the ones more likely to drive divergent views on intake [29,31,46], which was not absolutely accounted for in the current study design.

## 5. Conclusions

Inclusion of brown rice within mixed dishes is a potential means to overcome all of the key barriers to acceptability that limits its dietary inclusion. However, texture appears to not be masked within most of the dishes tested. Inclusion of brown rice within mixed dishes could be considered when targeting increased intake of whole grains at a population level or developing highly acceptable wholegrain food products (e.g., ready meals). The study design applied here provides a means of testing a wider range of refined grain and wholegrain ingredients in future studies, particularly with dishes that are culturally relevant to the target population.

## Figures and Tables

**Table 1:** Demographic detail of participants that took part in the sensory evaluation comparing brown and white rice dishes.

Dish (*n*-Value)	% Female (*n*-Value)	Median Age (Range) y	Frequency of Rice Consumption (% >3 Times/wk:<3 Times/wk)	% Habitual Rice Choice (BR:WR:Both)
Garlic Rice (29)	39.3 (11)	25 (20–58)	63.3:36.7	3.6:67.9:28.6 *
Rice and Beans (32)	43.8 (14)	22 (18–59)	78.1:21.9	10.7:71.4:17.9
Jollof Rice (28)	64.2 (18)	30.5 (21–43)	76.9:21.1	3.6:78.6:17.9
Rice Pudding (30)	53.3 (16)	25.5 (20–62)	75.9:24.1 *	10.0:66.7:23.3
TOTAL (93)	52.7 (48)	25 (18–62)	73.3:26.7 *	7.6:71:7:20.7 *

Total of 93 participants includes individuals that took part in tasting multiple dishes. * Includes non-responses.

**Table 2:** Comparison of acceptability of rice dishes across four different characteristics by participants.

Dish	Appearance (Median (LQ-UQ))			Aroma (Median (LQ-UQ))			Taste (Median (LQ-UQ))			Texture (Median (LQ-UQ))		
	White	Brown	*p*-Value	White	Brown	*p*-Value	White	Brown	*p*-Value	White	Brown	*p*-Value
Garlic Rice	4 (4–5)	3.5 (3–4)	0.006	4 (3.25–5)	3 (3–4)	0.012	4 (3.25–5)	4 (3–4.75)	0.608	4 (4–5)	3 (2.25–4)	<0.001
Rice and Beans	4 (4–4.75)	4 (4–5)	0.805	4 (3.25–5)	4 (3–5)	0.588	4 (4–5)	4.5 (4–5)	0.306	4 (4–5)	4 (4–5)	0.614
Jollof Rice	5 (4–5)	4 (3–4.75)	0.002	4 (4–5)	4 (3–4)	<0.001	4 (4–5)	3 (3–4)	<0.001	4 (4–5)	3 (2–4)	0.001
Rice Pudding	3 (3–4)	3 (2–4)	0.924	4 (3–4)	4 (3–4)	0.314	4 (3–5)	4 (2–4)	0.0136	4 (3–5)	3 (2–4)	0.196

*n* = 29 participants took part in the Garlic Rice sensory evaluation, *n* = 32 for Rice and Beans, *n* = 28 for Jollof Rice, and *n* = 30 for Rice Pudding. A *p*-value of <0.05 was considered statistically significant for Wilcoxon Ranked Sign tests.

**Table 3:** Qualitative themes shared by study participants related to the acceptability of white rice and brown rice alternatives for the rice dishes tested.

	White Rice		Brown Rice	
Characteristic	Themes	Exemplar Quotes	Themes	Exemplar Quotes
Appearance	Familiarity	“As expected appearance…” (F25, GR)] “Looks like I made it myself” (M23, RB) “…looked like rice pudding [from] when I was a child” (F21, RP)	Less preferable	“Not something I would choose over white rice” (M20, GR) “It looked overcooked as it was darker …” (F21, RB) “Color and presentation not so appealing” (F37, JR)
	Appealing	“Fine white colour and [was] unclumpy [sic]” (M34, GR) “Looked attractive, [with] bright colours from the accompanying vegetables/beans” (F44, RB) “Looks bright and flavourful” (M26, JR) “Attractive from first appearance” (M30, RP)	Comparable	“Same as [white rice sample], looks appetizing” (M21, RB) “Looks almost identical as [other sample]” (M24, RP)
			Better	“Better colour contrast with vegetables…” (M31, RB)
Aroma	Familiarity	“How I’d expect” (M31, GR) “Smelled familiar [, of] cinnamon maybe?” (F24, RP) “Aroma was inviting…” (F40, JR) “…reminded me of Christmas” (F21, RP)	Less preferable	“Not unpleasant but also not inspiring” (M53, GR) “Didn’t smell as fragrant” (M21, RB) “Not [the] expected aroma” (F37, JR) “Smelled bready… [like] wholegrain, not sweeter (M21, RP)
Taste	Familiarity	“Tastes like takeaway…” (M31, GR) “I like the spicy flavour” (F22, JR) “The taste is familiar” (F26, JR)	Comparable	“Like the last [sample], I thoroughly enjoyed it” (M23, RB) “I can’t really “get” the taste” (F29, JR)
	Appealing	“Rice packed with flavours of the other ingredients used” (F24, RB) “Tasted great, not too sweet. Very tasty” (M20, RP)	Less preferable	“Too hard to chew” (F33, JR) “Much dryer and grainier” (M21, RB) “Texture is okay but [would] like it softer” (F33, RP) “Appears to have particles after chewing” (M34, GR)
Texture	Familiarity	“As expected for rice” (M53, GR) “The taste was nice and the sort of food, I eat” (F44, RB) “Similar to what I consume” (F30, RP)	Better	“It is not bland or “water” tasting like some rice” (F22, GR) “Nice[r] taste than [the white rice sample]” (F21, RB) “The taste was better than expected” (F23, JR)
	Pleasanttexture	“Perfectly moist and not dry” (M24, JR) “Better grain separation” (F42, RB) “It’s softer and easy to eat” (F23, RP)		

Participant sex (M = male, F = female) and age (y) and sex presented with each exemplar quote, alongside detail of rice dish consumed (GR = Garlic Rice, RB = Rice and Beans, JR = Jollof Rice, RP = Rice Pudding).

## Data Availability

Original, anonymized data are available upon request.

## Appendix A

Please see below for ingredients list and questionnaires used in the current study.

### Appendix A.1. Ingredients Lists Used in All Dishes (Shared with Participants)

The ingredients used for the three rice recipes in this study are vegan, gluten-free, and dairy-free. If you are allergic or intolerant to any of these ingredients, you should not take part in this study.

The ingredients used in the rice recipes are as follows:

**Rice & Beans**
Ingredients:
  - White/brown rice (basmati)
  - Onion
  - Garlic
  - Paprika
  - Canned red kidney beans
  - Tomatoes
  - Green/red/yellow pepper
  - Lemon
  - Pepper powder
  - Sea salt
**Rice Pudding**
Ingredients:
  - White/brown rice (basmati)
  - Almond milk
  - Vegan butter
  - Brown sugar
  - Vanilla extract
  - Ground cinnamon
  - Lemon
  - Salt
**Garlic Rice**
  - White/brown rice (basmati)
  - Onions
  - Garlic
  - Olive oil
  - Salsa
  - Sea salt
**Jollof Rice**
Ingredients:
  - White/brown rice (basmati)
  - Onions
  - Garlic
  - Sunflower oil
  - Chopped tomatoes
  - Salt
  - Curry powder
  - Thyme
  - Bay leaf
  - Carrot
  - Spring Onion
  - Fresh Tomatoes
  - Vegetable stock cubes
  - Black pepper

### Appendix A.2. Wholegrain Food Sensory Questionnaire

1. In front of you is one sample (A). Taste the sample and tick (✓) how much you like or dislike each of the characteristics. **You can taste the sample more than once.**

	**Appearance**	**Aroma**	**Taste**	**Texture**
Like very much				
Like				
Neither like nor dislike				
Dislike				
Dislike very much				
Unwilling to try				

2. Why did you give the score above for appearance?

3. Why did you give the score above for aroma?

4. Why did you give the score above for taste?

5. Why did you give the score above for texture?

1. In front of you is one sample (B). Taste the sample and tick (✓) how much you like or dislike each of the characteristics. **You can taste the sample more than once.**

	**Appearance**	**Aroma**	**Taste**	**Texture**
Like very much				
Like				
Neither like nor dislike				
Dislike				
Dislike very much				
Unwilling to try				

2. Why did you give the score above for appearance?

3. Why did you give the score above for aroma?

4. Why did you give the score above for taste?

5. Why did you give the score above for texture?

**Thank you for your collaboration**
